# Miao sour soup influences serum lipid via regulation of high‐fat diet‐induced intestinal flora in obese rats

**DOI:** 10.1002/fsn3.3136

**Published:** 2022-12-07

**Authors:** Qianqian Zhou, Zihan Qu, Nanlan Wang, Huijuan Liu, Hongmei Yang, Huiqun Wang

**Affiliations:** ^1^ School of Public Health, the Key Laboratory of Environmental Pollution Monitoring and Disease Control, Ministry of Education Guizhou Medical University Guiyang China; ^2^ Guizhou provincial Center For Disease Control And Prevention Guiyang China; ^3^ Guizhou Food Nutrition and Health Engineering Research Center Guiyang China

**Keywords:** high‐fat diet, intestinal flora, Miao sour soup, obesity

## Abstract

Obesity is associated with the gut microbiota and has been shown to cause gut microbiota disturbances. Our previous studies have demonstrated that Miao sour soup (SS) contains abundant short‐chain fatty acids (SCFAs) which can be used as energy substrates of intestinal flora to selectively stimulate their growth and reproduction. Therefore, we explored whether the intestinal microbiota of rats with high‐fat diet‐induced obesity could be restored to normal by SS intervention. Male obese rats were divided into five groups randomly after successful modeling of obese rats: normal diet, high‐fat diet (HDF), HFD + SS, HFD with antibiotic, and HFD with antibiotic + SS. After 12 weeks of intervention, the weight and serum lipid of obese rats decreased. Furthermore, 16S rRNA analysis showed an imbalance and a decrease in the abundance and diversity of intestinal flora in obese rats, which improved after SS intervention. At the phylum level, Firmicutes increased while Proteobacteria decreased. The composition of the intestinal flora recovered at the genus level, inhibiting the reproduction of pathogenic bacteria, while the levels of SCFA‐producing bacteria such as *Blautia* and *Lactococcus* and the levels of SCFAs in cecal contents increased. In addition, SS reduced the levels of TNF‐α and IL‐6 in the intestinal mucosa of obese rats, increased the contents of PYY and GLP‐1 in colon tissue, and increased the expression of tight junction protein Occludin and ZO‐1 in the intestinal epithelium. Taken together, SS can regulate the intestinal flora of obese rats and improve the intestinal flora to facilitate weight loss and lipid reduction.

## Graphical abstract

Obesity is a health condition of increasing importance. Obesity has been associated with an imbalance in the intestinal flora. Miao sour soup (SS) contains abundant short‐chain fatty acids (SCFAs), which can be used as energy substrates to selectively stimulate the growth and reproduction of intestinal flora. This study aims to determine whether SS intervention could restore the imbalanced intestinal flora of rats with high‐fat diet‐induced obesity. We found improvements in the abundance and diversity of the intestinal flora, and a significant reduction in weight and serum lipid levels following intervention with SS. We believe that our study makes a significant contribution to the literature because our findings demonstrate the therapeutic potential of dietary intake of SS in achieving weight loss and reducing serum lipid levels, thus ameliorating the effects of obesity induced by high‐fat diet.

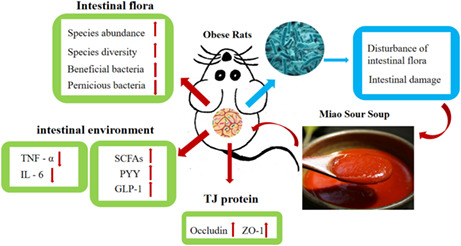

## 1 INTRODUCTION

Obesity is a chronic metabolic disease that results in excessive accumulation and/or abnormal distribution of fat in the body and is associated with significant health hazards. Over the last two decades, the prevalence of adults with an overweight BMI increased by 27.5% (Ng et al., 2014). There has been a significant and well‐documented increase in the prevalence of obesity around the world since the 1980s, which continues to rise even today (Inoue et al., 2018). The intake of high‐sugar and high‐fat diets often leads to a long‐term imbalance in energy intake and energy expenditure (Hall et al., 2012), which leads to many chronic noncommunicable diseases, such as hypertension, diabetes, coronary heart disease, myocardial infarction, and related complications. Currently, measures to control obesity, including drug, behavioral, and surgical interventions lack efficacy (Ivezic‐Lalic et al., 2013). Studies have found that the excess energy generated by the intake of high‐calorie diets including high‐fat and high‐sugar diets is one of the biggest risk factors for the occurrence and development of obesity (Xia et al., 2022). Dietary interventions and treatments may be beneficial in improving obesity (Esmaeili et al., 2022; Wang et al., 2022). Obesity caused by a high‐fat diet (HFD) leads to changes in intestinal microbial composition, as well as decreases microbial diversity and changes in specific bacterial classification (Miyamoto et al., 2019). Besides, the maladjustment of gut flora may also contribute to obesity, lipid metabolism disorders and related diseases (Liu et al., 2021; Nyangale et al., 2012).

The gut microbiota plays an important role in the gastrointestinal tract of the human body by regulating the absorption and metabolism of nutrients, modulating the immune system, and producing vitamins (Aoki et al., 2016). Intestinal flora can also secrete short‐chain fatty acids (SCFA) to reduce intestinal pH, further killing foreign bacteria and inhibiting their secretions (Lynch & Pedersen, 2016). Butyric acid in SCFAs can also protect the integrity of intestinal mucosa by promoting the secretion of antimicrobial peptides. Combined with GPR43, SCFAs can promote the secretion of leptin, which can reduce food intake, increase energy release, inhibit the formation of fat cells, and consequently reduce body weight (Perez‐Burgos et al., 2013). SCFAs can also promote the secretion of insulin, improve the body's sensitivity to insulin, increase energy expenditure, and reduce fat accumulation. In addition, the combination of SCFAs and GPR109A induces the differentiation of Treg cells and IL‐10‐secreting T cells, which play an important role in the anti‐inflammatory process (Singh et al., 2014). *Bifidobacteria* in the intestinal flora can increase the number of CD4^+^ Treg cells in the intestinal tract as well as the secretion of IL‐10, effectively reducing intestinal inflammation and maintaining intestinal homeostasis (Moya‐Perez et al., 2015). *Lactobacillus* is a beneficial bacteria, which can strengthen the intestinal barrier function and regulate inflammation by inhibiting the adsorption and engraftment of some pathogenic bacteria, enhancing phagocytosis, stimulating the production of cell factors, and maintaining the balance of intestinal flora. *Lactobacillus* can also reduce the body fat percentage, and maintain blood glucose levels (Wang et al., 2019). Studies have found that *Lactobacillus* in yogurt can prevent certain proinflammatory factors from entering the blood, which not only plays an anti‐inflammatory role but also helps obese patients improve glucose metabolism by reducing postprandial blood glucose levels (Pei et al., 2017, 2018). Therefore, intestinal flora is closely related to the occurrence and development of metabolic diseases such as obesity.

However, many factors can affect the balance of the intestinal flora, leading to changes in the number and proportion of the intestinal flora. Turnbaugh et al. (2006) have shown that the structure of the mouse gut microbiota changes in response to a HFD. Some by‐products produced during the process of lipid metabolism, such as secondary cholic acid and hydrogen sulfide, damage the mucosa of the intestine, leading to mucosal inflammation and damage to the microenvironment in which the bacteria live. When researchers (Zhang et al., 2010) fed mice with the same genotype, they found that the intestinal microbiota of mice fed with a plant diet differed by about 60% from those fed with a HFD. When mice with different genotypes were fed the same type of diet, the difference in intestinal microbiota structure was only 12%. Studies have found that fermented foods can increase the diversity and species abundance of the intestinal flora of the host and reduce the inflammatory response. Thus, fermented foods have a potential role in reducing weight and fat by regulating intestinal flora.

Miao sour soup (SS) is a traditional fermented food of the Miao and Dong ethnic groups in southeastern Guizhou, China. The traditional process includes fermenting tomato and red pepper into semifinished products, adding ginger, Kizuko, and other raw materials for a second natural fermentation to produce *Lactobacilli*, *Acetobacter*, *Leuconostoc*, and yeast as well as beneficial bacteria such as *Mycobacterium* and *Lactobacillus*. Miao SS also contains abundant SCFAs, such as acetic acid, propionic acid, and butyric acid, which can be used as energy substrates to selectively stimulate the growth and reproduction of the intestinal flora (Perez‐Burgos et al., 2013). In the preliminary experiment, we found that SS regulates lipid metabolism. After SS intervention in obese rats, weight, serum lipid, and fat accumulation decreased. SS can also modulate the AMPKα signaling pathway, which reduces the synthesis of endogenous fatty acids in the liver of rats, thus playing a role in regulating lipid metabolism. Yet, most of the research only focuses on preparation technology, determination of nutritional composition, and molecular biology of SS. However, the relationship between SS and intestinal flora as well as its underlying mechanism in improving obesity has not been fully explored.

Therefore, this study aims to explore whether SS, a fermented food, could improve and normalize the abundance and diversity of intestinal microflora induced by HFDs in obese rats. This will enable the development of therapeutic interventions to restore the intestinal microbiota disturbances caused by obesity and other diseases.

## 2 METHODS

### 2.1 Preparation of Miao sour soup

SS was made from red pepper, tomato, salt, ginger, *Litsea pungens*, and other materials through natural fermentation, and was produced by Guizhou Mingyang Food Co., Ltd. (Guiyang, China). The SS stock solution was diluted with distilled water to a concentration of 0.8 g/ml, and heated to a boil. The SS mixture was then filtered using six layers of gauze to obtain the filtrate and boiled it following the usual preparation procedure. The filtrate was obtained for use in subsequent experiments.

### 2.2 Animal model

All animal testing in this study was conducted at the animal facilities of Guizhou Medical University Animal Center and complied with the standards in the Guide for the Care and Use of Laboratory Animals of China. Eight‐week‐old male Sprague Dawley (SD) rats (weighing 180 ± 20 g) were purchased from Guizhou Medical University Animal Center (Guizhou, China). All rats were fed in a room with controlled temperature (23 ± 1°C), relative humidity (50% ± 5%), lighting (12 h light/dark cycle), and were given free access to water and standard rodent chow. After 1 week of acclimatization, rats were randomly assigned to different experimental groups.

During the modeling period, rats were randomly divided into two groups: normal diet group (ND) (*n* = 10) and the HFD group (HFD1) (*n* = 40). High‐fat feed (D12451) and common‐standard feed (D12450B) were purchased from Biopike Biotechnology Co., Ltd. (Chengdu, China). After 12 weeks of diet induction, the body weight of rats in the two groups was measured. When the body weight of the HFD1 group was 20% higher than that of the ND group, it indicated the successful establishment of the obese rat model. Subsequently, the rats in the HFD1 group were further randomly divided into four intervention groups (*n* = 10 in each group): HFD group (HFD), HFD group with 8 g/kg of body weight (b.w.)/d SS (SSI), HFD group with antibiotics in drinking water (HFD + ABS), HFD group with antibiotics in drinking water and gavage with 8 g/kg b.w./d of red SS (HFD + ABS + SS). The experiment was completed after 12 weeks of intervention. The following antibiotics were added to the drinking water of the rats: 0.5 g/L vancomycin, 1 g/L neomycin sulfate, 1 g/L metronidazole, and 1 g/L ampicillin (Wuhan, China). The rats were given anesthesia by intraperitoneal injection of pentobarbital sodium after 12 h of fasting at the end of the experiments. Blood was collected from the heart by cardiac puncture. Serum was obtained by centrifuging the blood at 3000 rpm for 15 min and stored at −20°C. The adipose index and Lee's index of the rats were calculated using the following formula:

$$\begin{array}{c} \text{Adipose index}=\text{intra}-\text{abdominal}\;\mathrm{fat}/\text{body weight}\\ \;\times 100\;\left(\mathrm{Hao}\;\mathrm{et}\;\mathrm{al}.,\;2015\right). \end{array}$$

$$\begin{array}{c} {\mathrm{Lee}}^{’}s\;\text{index}=\text{body weight}\;{\left(g\right)}^{1/3}/\text{nose}-\text{to}-\text{anus length}\;\left(\mathrm{cm}\right)\\ \;\times 1000\;\left(\text{Novelli}\;\mathrm{et}\;\mathrm{al}.,\;2007\right). \end{array}$$

### 2.3 Blood sample analysis

The serum levels of TG, TC, LDL‐C, and HDL‐C were measured by an HD‐F2600 automatic biochemical analyzer from Jinan Hanfang Medical Instrument Co., Ltd. (Shandong, China).

### 2.4 Testing of 16S rRNA

Cecum contents of mice from each group were immediately frozen on dry ice and sent for 16S rRNA sequencing.

Extraction of genomic DNA: Next‐generation sequencing of the gut microbiota was performed using an Illumina platform (Anoroad Genome Technologies, Beijing, China). DNA from the cecum contents was extracted using a modified process in which 100 μl of phosphate‐buffered saline (PBS) was added to 100 μl of supernatant before extraction with EZ1 Advanced XL, and eluted in aliquots of 200 μl. After genomic DNA extraction, the extracted genomic DNA was detected by 1% agarose gel electrophoresis.

For bacterial DNA V3 variable region PCR amplification, the primers 338F/533R were used, and barcodes were synthesized according to the designated sequencing region. PCR was performed using TransGenAP221‐02 TransStartFastpfu DNA Polymerase on an ABI GeneAmp® 9700 instrument.

#### 2.4.1 Fluorescence quantification

Based on the preliminary electrophoresis results, the PCR products were quantified with a QuantiFluorTM‐ST Blue Fluorescence System (Promega) and then mixed according to the sequencing amount in each sample.

#### 2.4.2 MiSeq library construction

Magnetic beads were used to remove the self‐ligated fragments of the adaptors and then the library template was enriched by PCR amplification. Single‐stranded DNA fragments were produced by sodium hydroxide denaturation.

#### 2.4.3 Next‐generation sequencing

Next‐generation sequencing of the gut microbiota was performed using the instrument of Annoroad Gene Technology Co., Ltd. Only reads with a final length of more than 110 bp were reserved for downstream analysis. The number of high‐quality sequences used for taxonomical assignment ranged from 36,220 to 67,489 per sample. The filtered sequences were then processed using the Trimmomatic tool. The resulting amplicon sequence variants (ASVs) were used for diversity analyses (alpha and beta) and taxonomic classification. The Bayes classifier was trained on V3 region sequences from Silva Release 128, RDP Release 11.1, and Greengenes Release 13.5 (97% identity for operational taxonomic units [OTUs] using Usearch [version 7.1, http://drive5.com/uparse/]).

### 2.5 Measurement of TNF‐α, IL‐6, PYY, and GLP‐1 in intestine tissue

One part of the intestine tissue and nine parts of PBS solution was placed in a small beaker. The tissue was immediately cut into pieces with ophthalmic scissors and poured into an Eppendorf (EP) tube. The tissue was ground for 5 min to homogenize. The EP tube was centrifuged at 3000 rpm for 20 min and then the supernatant was removed and placed into different EP tubes. The levels of TNF‐α, IL‐6, PYY, and GLP‐1 in colon tissue were determined by enzyme‐linked immunosorbent assay (R&D Systems, Minneapolis, MN, USA).

### 2.6 Detection of SCFAs levels in intestinal contents

The intestinal contents of the cecum segment were stored in an enzyme‐free biochemical tube and cryopreserved at −80°C. The concentrations of acetic acid, propionic acid, *n*‐butyric acid, and *n*‐valeric acid in the intestinal contents were determined using a meteorological chromatograph. The conditions for gas chromatography are as follows: sample volume, 1 μl; inlet temperature, 220°C; column flow rate, 0.95 m L/min; column temperature, 90°C, equilibrium time, 0.5 min, temperature rise, 150°C at 5°C/min; retention time, 7 min; detector temperature, 230°C; hydrogen flow, 40 ml/min; airflow, 4000 ml/min; and tail blowing volume, 40 ml/min.

### 2.7 Western blot analysis

The frozen intestinal tissues were washed and homogenized on ice with lysis buffer and centrifuged at 13,000 rpm for 30 min at 4°C. The supernatant was diluted and the protein content was measured by a BCA protein quantification kit. Proteins were separated by sodium dodecyl sulfate polyacrylamide gel electrophoresis and transferred to a polyvinyl difluoride membrane. The blots were probed with primary antibodies against Occludin and ZO‐1 (Shanghai Fusheng Industria, Shanghai, China) and GAPDH (Hangzhou Fude Biotechnology, Hangzhou, China), followed by a secondary antibody. Kodak X‐ray film was used for compression, development, fixation, scanning, and data analysis.

### 2.8 Statistical analysis

Bacterial diversity was estimated by richness calculation (number of OTUs) and Shannon and Simpson diversity indices. Simca software (Umetrics, Umea, Sweden) was used to draw principal component analysis (PCA) maps of the intestinal microbiota. GraphPad Prism (GraphPad Software, USA) and Photoshop (Adobe, USA) were used to draw phylum and genus level heat maps of intestinal microbiota as well as bar charts.

All statistical analyses of the experimental data were performed with SPSS (version 23.0; SPSS, Inc., Chicago, IL, USA). Data were subjected to equal variance and normality testing (Kolmogorov–Smirnov test). All quantitative data were expressed as mean ± standard deviation. When the variances were uniform, the data of multiple groups were compared using a completely random design analysis of variance (one‐way ANOVA), and the pairwise comparisons between groups were performed using the LSD (least significant difference) method. The correlation analysis between indicators was conducted using the Pearson's correlation test. A *p* < .05 was considered significant.

## 3 RESULTS

### 3.1 HFD‐induced obesity

**FIGURE 1:**
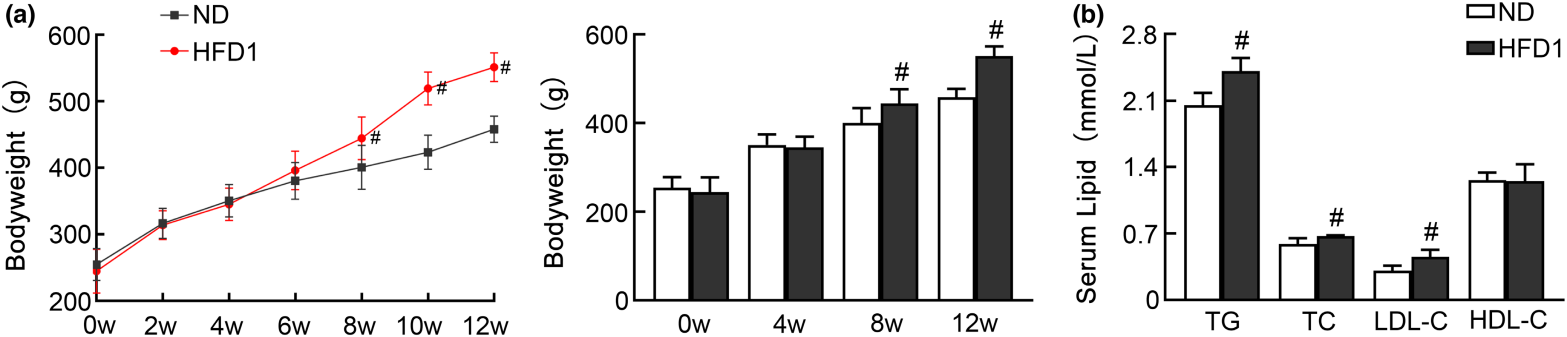
Establishment of obese rats model. Rats were fed a normal diet (ND) and a high‐fat diet (HFD) for 12 weeks and their (a) body weight and (b) blood fat were measured. ^#^ *p* < .05 versus ND group.

Rats were fed with an HFD and their body weight and blood fat were analyzed at 12 weeks. As shown in Figure 1, the body weight of the HFD group (551.67 ± 21.63) was 20% higher than that of the ND group (458.22 ± 19.59) (*p* < .05), and the serum levels of TG, TC, and LDL‐C in the HFD group were significantly higher than in the ND group (*p* < .05), indicating the successful establishment of HFD‐induced obesity.

### 3.2 SS reduces body weight and improves serum lipid levels in obese rats

**FIGURE 2:**
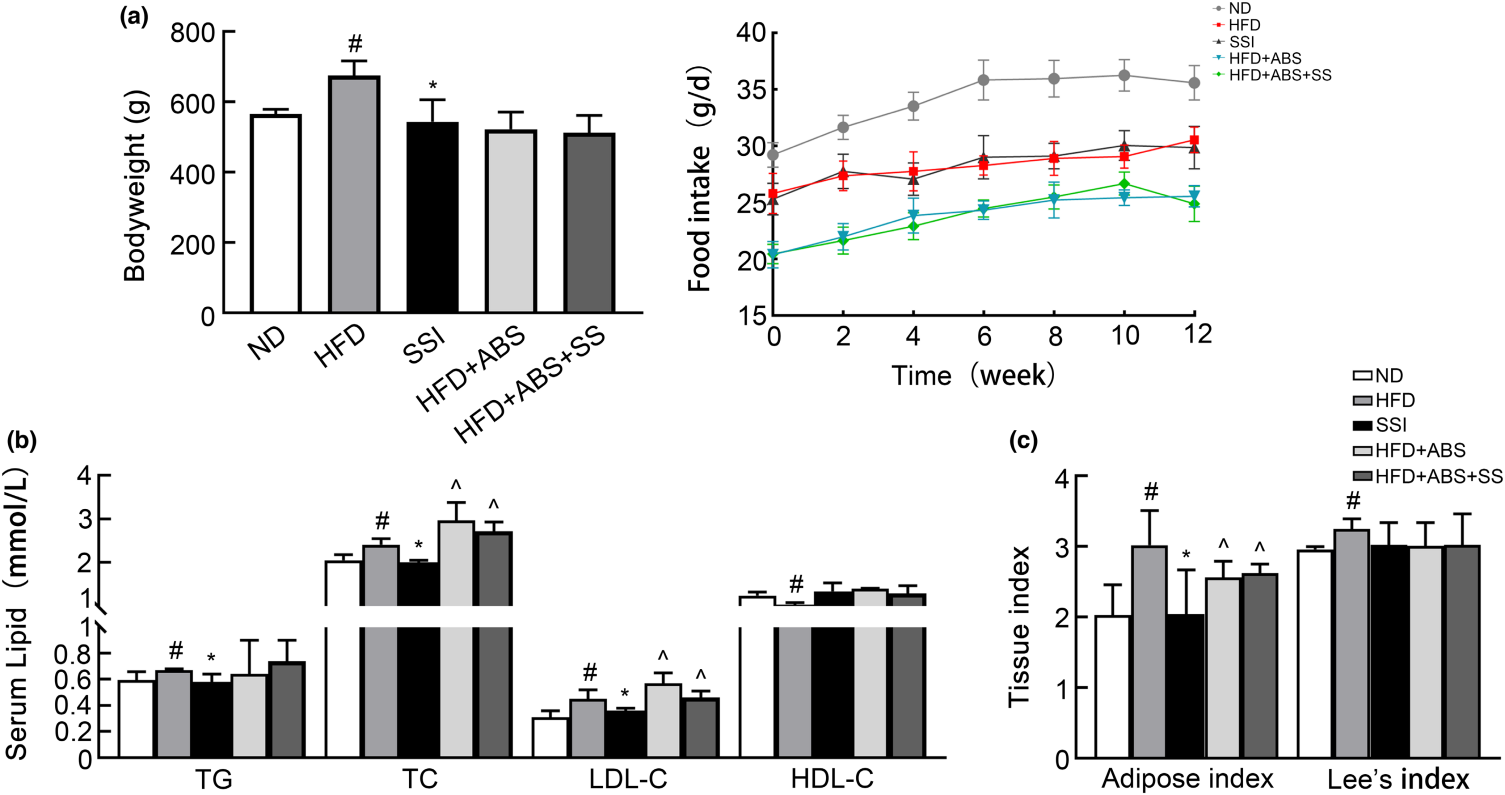
Effects of SS intervention on body weight, food intake, serum lipid, and tissue index in obese rats. (a) Body weight and food intake, (b) serum lipid levels, and (c) tissue index of rats in the five groups after 12 weeks of intervention. ^#^ *p* < .05 versus ND group. *p < .05 versus HFD group. ^^^ *p* < .05 versus SSI group.

As shown in Figure 2, the body weight of rats in the SSI group was lower than that of the HFD group (*p* < .05), indicating that SS could inhibit weight gain in obese rats. In addition, the serum concentrations of TG, TC, and LDL‐C in the SSI group were significantly lower compared to the HFD group (*p* < .05), indicating an effective improvement in the serum lipid level of rats in the SSI group. The total energy intake of a HFD in the HFD group and SSI group was equal, but were higher than that in the ND group. Compared to the SSI group, serum TG, TC, and LDL‐C levels were increased (*p* < .05) in the two groups supplemented with antibiotics, but food intake and body weight were decreased. The coefficient of fat of the obese rat group was higher than that of the ND and SSI groups (*p* < .05), and Lee's index was also higher than that of the ND group, which decreased after the intervention with SS.

### 3.3 Alpha diversity analysis based on 16S rRNA gene sequencing

**FIGURE 3:**
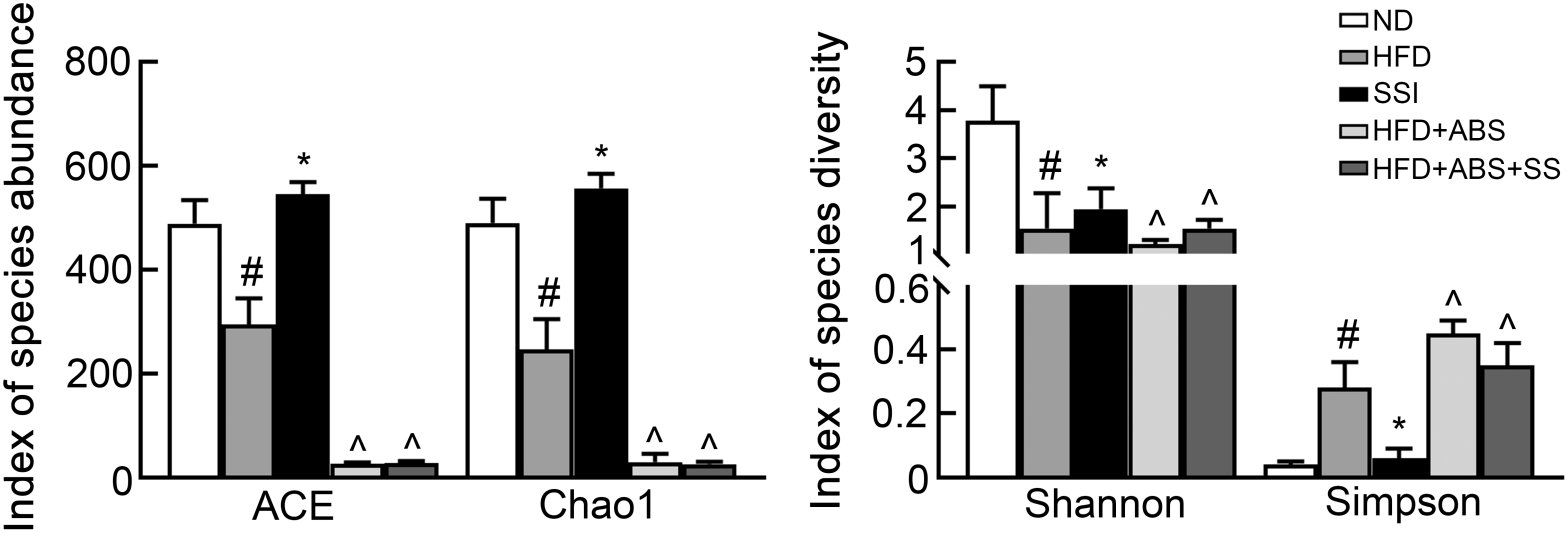
Analysis of alpha diversity among five groups of rats. ^#^ *p* < .05 versus ND group. *p < .05 versus HFD group. ^^^ *p* < .05 versus SSI group.

Figure 3 shows that compared with obese rats, the ACE index and Chao1 index of rats in the SSI group were increased (*p* < .05), indicating that SS can improve the decreased abundance of species in the intestinal microflora caused by a HFD. At the same time, compared to the obese rats, the Shannon index was increased and the Simpson index was decreased in the SSI group (*p* < .05), suggesting that SS can improve the diversity of intestinal microflora in obese rats.

### 3.4 PCA of intestinal flora in rats

**FIGURE 4:**
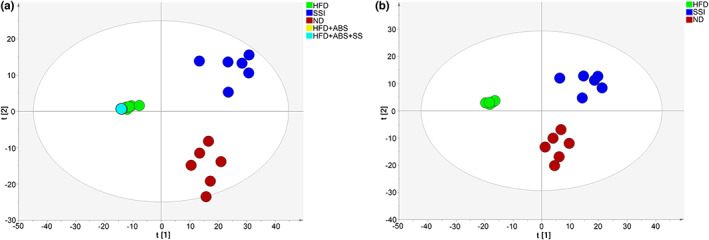
Response of the intestinal flora to a high‐fat diet in the Miao sour soup (SS) group. Score plots of PCA in rat intestinal flora between (a) the HFD, SSI, ND, HFD + ABS, and HFD + ABS + SS groups (*R* ^2^ *X* = 0.438, *R* ^2^ *Y* = 0.137) and (b) the HFD, SSI, and ND groups (*R* ^2^ *X* = 0.356, *R* ^2^ *Y* = 0.171).

Figure 4a is the PCA of the five groups. Due to the destruction of the intestinal flora by broad‐spectrum antibiotics, the distribution of the intestinal flora in the two groups of mice that received additional gavage antibiotics tends to be unanimous. In Figure 4b, a more similar distribution of the intestinal flora of obese rats after SS intervention and the ND group of rats was observed.

### 3.5 Intervention of SS can improve intestinal flora of obese rats

**FIGURE 5:**
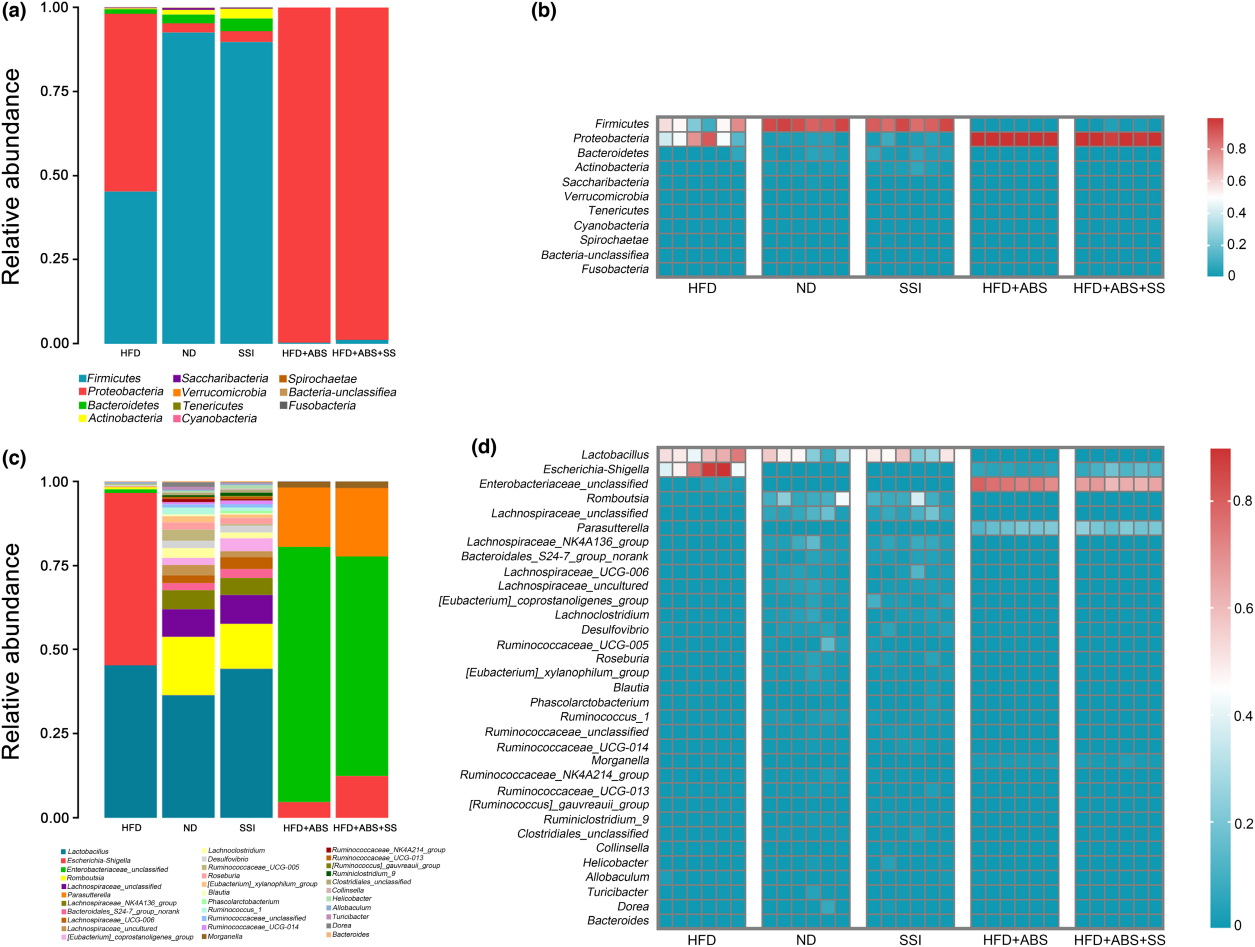
Changes in the composition and content of intestinal microflora in the phylum and genus level of rats in the five groups. (a) Phylum level bar chart of intestinal flora. (b) Phylum level heat map of intestinal flora. (c) Genus level bar chart of intestinal flora. (d) Genus level heat map of intestinal flora.

As can be seen from Figure 5, the composition of intestinal flora was dominated by Firmicutes, Proteobacteria, Bacteroidetes, and Actinobacteria at the phylum level. The content of Firmicutes was the lowest in the two groups treated with antibiotics, and was higher in the SSI group than in the HFD group, indicating that the SS could restore the content of Firmicutes in the obese rats. The content of Proteobacteria in the SSI group was lower than that in the HFD group and the antibiotic intervention groups, and was similar to that in the ND group, indicating the inhibitory effect of SS on the intestinal Proteobacteria. The levels of Bacteroidetes and Actinomycetes in the SSI group were higher than in the HFD group and similar to the ND group. This can also be seen from the phylum level heat map.

There were also differences in the composition of intestinal flora in each group at the genus level. The contents of *Lactobacillus* in the HFD, ND, and SSI groups were comparable, but the abundance of *Escherichia_Shigella* in the HFD group was higher than that in the ND group and SSI group. The structure of the *Lachnospiraceae_g_unclassfied*, *Lachnospiraceae_UCG_006, Lachnospiraceae_NK4A136_group*, *Romboutsia*, and other bacteria were similar in the SSI and ND groups. The results showed that SS could improve the composition of intestinal flora, increase the number of beneficial bacteria, inhibit pathogenic bacteria, and restore the damaged intestinal ecology of obese rats to a healthy state at the genus level. This is also well reflected on the heat map at the genus level.

### 3.6 SS can affect the intestinal environment of obese rats

**FIGURE 6:**
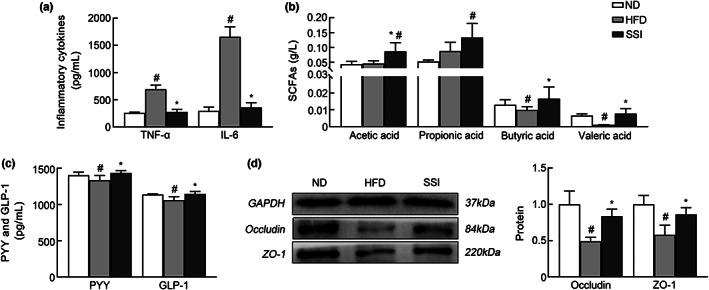
The intestinal environment of rats in the five groups. (a) Intestinal mucosal inflammatory factors in the intestinal mucosa. (b) The content of short‐chain fatty acids. (c) PYY index and GLP‐1 index in colon tissue. (d) Expression of tight junction protein in intestinal mucosa epithelium. ^#^ *p* < .05 versus ND group. *p < .05 versus HFD group.

As can be seen from Figure 6, the levels of the intestinal mucosal inflammatory factors, TNF‐α and IL‐6, were increased in the HFD group (*p* < .05) compared to the normal group, while TNF‐α and IL‐6 levels in obese rats decreased after SS intervention (*p* < .05). At the same time, compared with the ND group, the contents of PYY and GLP‐1 in colonic tissue of rats in the HFD group were decreased (*p* < .05), and that of the SSI group were significantly upregulated after SS intervention (*p* < .05). In addition, compared with obese rats, the levels of acetic acid, butyric acid, and valeric acid in the intestinal contents of rats in the SSI group were increased significantly (*p* < .05), indicating that SS can alter SCFAs in the intestines. Compared with the ND group, the levels of intestinal epithelial tight junction proteins Occludin and ZO‐1 in obese rats were decreased (*p* < .05). After SS intervention, the protein levels of Occludin and ZO‐1 increased significantly (*p* < .05).

### 3.7 Correlation analysis

In the SSI group, the serum TG level was positively correlated with the levels of TNF‐α (*r* = .888, *p* < .05) and IL‐6 (*r* = .887, *p* < .05) in the intestinal mucosa, and was negatively correlated with the ACE index (*r* = −.835, *p* < .05) and Shannon index (*r* = −.944, *p* < .05). The serum TC level positively correlated with IL‐6 level (*r* = .835, *p* < .05) in intestinal mucosa, and negatively correlated with ACE (*r* = −.832, *p* < .05) and Shannon (*r* = −.913, *p* < .05) indices. The serum LDL‐C level was positively correlated with the TNF‐α level (*r* = .827, *p* < .05) in the intestinal mucosa, and was negatively correlated with the HDL‐C level (*r* = −.956, *p* < .05). The serum HDL‐C level was positively correlated with the ACE index (*r* = .815, *p* < .05). The ACE index was positively correlated with Chao1 index (*r* = .879, *p* < .05) and Shannon index (*r* = .923, *p* < .05). The Chao1 index was positively correlated with the Shannon index (*r* = .836, *p* < .05). Meanwhile, the Shannon index was negatively correlated with the levels of TNF‐α (*r* = −.833, *p* < .05) and IL‐6 (*r* = −.881, *p* < .05) in the intestinal mucosa.

## 4 DISCUSSION

In our previous experiments, we revealed for the first time that SS has a preventive effect on HFD‐induced obesity and can affect the levels of AMPK/SREBP pathway‐related genes and proteins to regulate lipid metabolism in obese rats (Yang et al., 2021). In this study, we also found that the serum TG, TC, and LDL‐C levels of rats were increased significantly following a HFD, whereas the aberrant serum lipid levels of these obese rats were effectively ameliorated after SS intervention. Moreover, the HFD and the energy intake of rats in the HFD group and SSI group were similar. This suggests that dietary changes do not influence the effect of SS on reducing obesity, which was consistent with previous experimental results. Of note, in this study, we found that a disturbed intestinal flora in the HFD group compared to the ND group, which improved effectively following SS intervention. We used broad‐spectrum antibiotics to disturb the intestinal flora of obese rats (Ma et al., 2007). We found that SS does not play a role in reducing lipid levels in disturbed intestinal flora. Moreover, the lipid levels of the two groups with the additional antibiotic intervention were higher than that of the SSI group. However, the rats in both groups lost weight, which may be associated with reduced food intake. Therefore, we speculated that SS could regulate lipid metabolism by reshaping the composition of the intestinal flora.

SS is a type of fermented food that constitutes tomato, red pepper, and ginger as the main raw materials, and is prepared by natural microbial fermentation. SS contains certain amounts of lycopene and capsaicin which can regulate serum lipid levels (Hwang et al., 2005; Mosqueda‐Solis et al., 2018; Vallverdu‐Queralt et al., 2015). In addition, Wastyk et al. proposed that fermented food can affect the intestinal microflora of the host by increasing the diversity of the intestinal microflora and reducing the inflammatory response of the host (Wastyk et al., 2021). We found that SS, a fermented food, has a similar effect on obese rats. In this experiment, we observed that SS can improve obesity and reduce serum lipid levels in rats by protecting the intestinal mucosal barrier. On one hand, SS strengthened both mechanical and immune barriers in obese rats. After the SS intervention in obese rats, the expression of the tight junction proteins ZO‐1 and Occludin in small intestinal epithelial cells increased. Tight junction protein can maintain the permeability and integrity of the intestinal barrier (Choi et al., 2017; Turner, 2009), and the increase in its expression level indicated that the intestinal epithelial structure of obese rats becomes compact after SS intervention. At the same time, the breakdown of tight junctions is usually accompanied by intestinal inflammation. The results showed that the levels of inflammatory cytokines IL‐6 and TNF‐α were decreased in the SS intervention group, indicating an improvement in the inflammatory response of obese rats. Therefore, SS can protect the mechanical and immune barrier of obese rats and facilitates the maintenance of healthy intestinal flora.

Moreover, SS can improve the chemical and biological barriers of obese rats. Many studies have shown that low microbiota diversity is associated with many chronic noncommunicable diseases, such as obesity and diabetes (Le Chatelier et al., 2013; Turnbaugh et al., 2009). In this experiment, we observed that the ACE index and Chao1 index of intestinal flora increased after SS intervention in obese rats, suggesting that SS can increase the species abundance of the intestinal flora. In addition, the Shannon index increased and the Simpson index decreased in the SSI group, suggesting improvements in the intestinal microbial diversity. The species abundance and diversity of intestinal flora in the two groups supplemented with antibiotics were the lowest, implying an imbalance in the intestinal flora as a result of the use of broad‐spectrum antibiotics.

In terms of intestinal flora, Firmicutes, Bacteroidetes, and Proteobacteria occupy the highest proportion at the phylum level. Among them, Firmicutes and Bacteroidetes are the two dominant groups and account for more than 90% of the human intestinal tract microbiome (Lozupone et al., 2012; Mark et al., 2017). As shown in Figure 4, compared with the SSI group, the content of Firmicutes and Bacteroidetes was significantly decreased in obese rats, while the content of Proteobacteria was increased. Following SS intervention, the level of Firmicutes and Bacteroidetes in obese rats was recovered, and the overall composition of the intestinal flora resembled that of the ND group. It is well established that patients with obesity have increased proportions of Firmicutes/Bacteroidetes, which can effectively stimulate the intestinal absorption of nutrients and calories. In this experiment, we found that the Firmicutes/Bacteroidetes ratio of rats in the HFD group was higher than that of rats in the SSI group, which was consistent with the findings of previous studies. The results showed that although SS did not reduce the content of Firmicutes, it significantly increased the content of Bacteroidetes, which reduced the ratio of Firmicutes to Bacteroidetes in the SSI group, reduced the absorption of food calories, and further affected the occurrence and development of obesity. *Lactobacillus* and *Enterococcus* are common bacteria of the phylum Firmicutes. *Bifidobacterium* and *Lactobacillus* have proven to have a direct relationship with cholesterol metabolism and can regulate serum lipid levels (Cani et al., 2008; Zhu et al., 2013). At the same time, some Bacteroidetes can also regulate lipid metabolism disorders induced by a HFD (Dumas et al., 2006). This suggests that SS can regulate lipid metabolism by restoring the levels of Firmicutes and Bacteroidetes.

Compared to the HFD group, SS could increase the abundance of *Blautia*, *Lactococcus*, and *Trillium*, at the genus level while reducing the abundance of *Shigella* and *Escherichia coli*. In Figure 5, the levels of SCFAs in the SSI group were significantly increased, which may be associated with some bacteria. *Blautia* and *Lactococcus* belong to the SCFA‐producing group and have a significant positive correlation with the SCFAs (Greetham et al., 2004; Park et al., 2012). SCFA‐producing bacteria can maintain the stability of the intestinal microecology by repairing mucosal damage caused by pathogens, providing energy for intestinal epithelial cells, and slowing down intestinal inflammation (De Filippo et al., 2010; Maslowski et al., 2009). At the same time, SS is rich in SCFAs such as acetic acid and lactic acid. They can also serve as the energy substrate for the intestinal flora, nourish intestinal epithelium, and improve healthy intestinal flora (Haenen et al., 2013). In addition, SCFAs can also regulate energy intake through the gut–brain axis, which can trigger satiety (Kimura et al., 2014). The experimental results showed that the contents of PYY and GLP‐1 in the colon tissues of rats in the SSI group were significantly increased after SS intervention. Studies have shown that SCFA can activate GPR41 to release PYY and GLP‐1 in the intestine. PYY and GLP‐1 can regulate intestinal cells to control gastric emptying and food intake, and stimulate insulin secretion to reduce blood glucose, which plays an important role in the prevention and treatment of obesity and diabetes. These results indicate that SS can increase the production of SCFAs and stimulate the secretion of PYY and GLP‐1 to ameliorate obesity, which is consistent with the study by Zhou et al. (2006). Therefore, while the intestinal flora influences the levels of SCFAs, the intestinal flora is also positively impacted by the SCFAs. It can be seen from these results that SS can increase the abundance of beneficial bacteria, potentially inhibit pathogenic bacteria, help regulate the imbalance of intestinal flora, and protect the intestinal mucosal biological barrier of obese rats. SS can also increase the secretion of SCFAs such as acetic acid to protect the intestinal mucosal chemical barrier of obese rats, thereby reducing weight gain and lipid levels.

**FIGURE 7:**
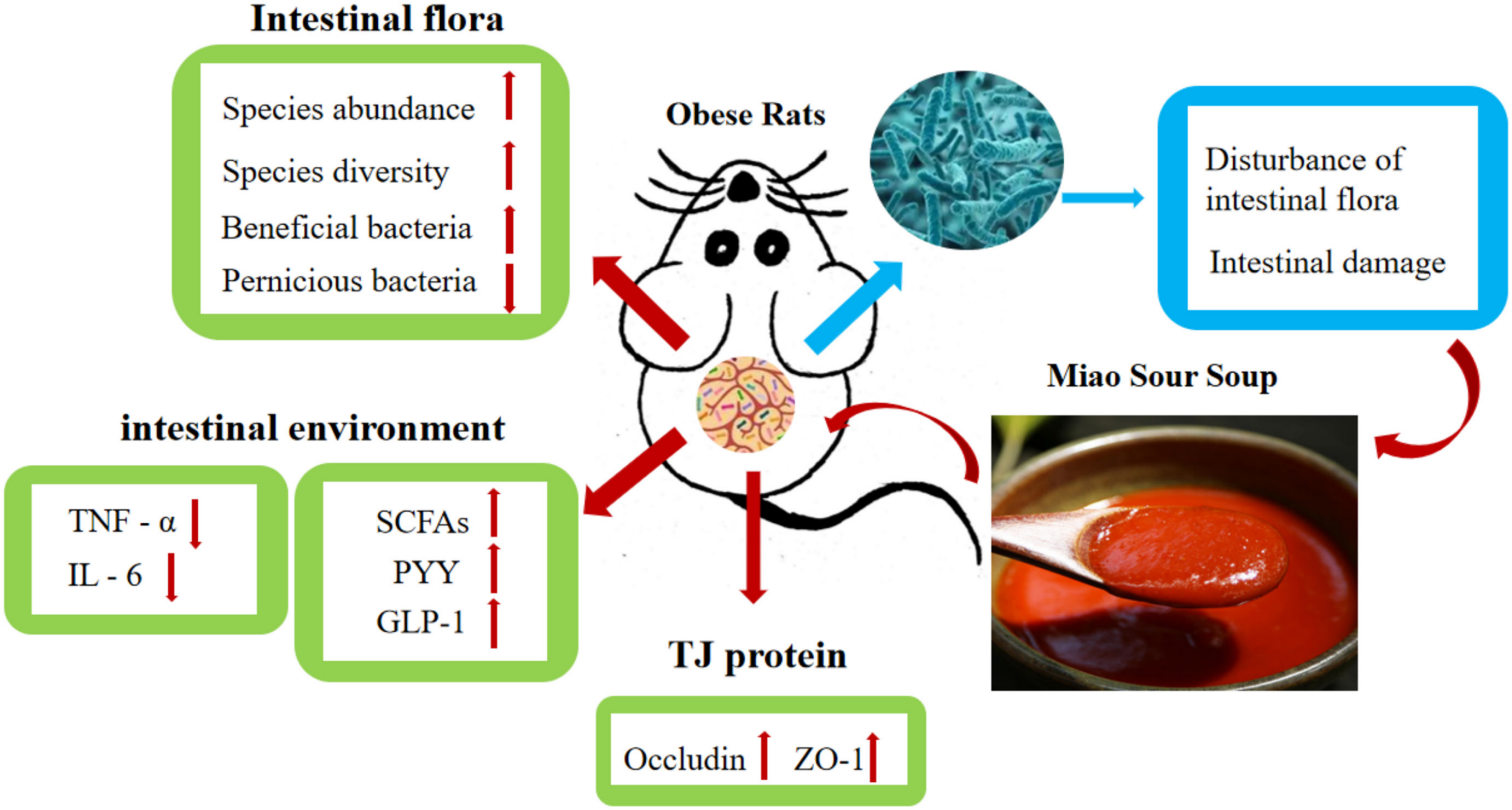
Summary: Effects of SS on intestinal flora and intestinal environment of obese rats.

Figure 7 shows the mechanism of SS intervention in obese rats. Above all, a HFD can lead to changes in the structure of the rat intestinal flora, reducing the content of beneficial bacteria such as *Lactobacillus* and *Bifidobacterium*. SS can increase the species abundance and diversity of intestinal flora, adjust the intestinal flora composition, reduce small intestine mucosal inflammation, and increase the expression of the tight intestinal epithelial protein in obese rats. In addition, the weight and serum lipid levels of obese rats were reduced. Our findings provide substantial evidence of the critical role of SS in reducing weight and lipids by improving the imbalance of the intestinal flora.

## 5 CONCLUDING REMARKS

Overall, our study findings suggest that SS can reduce weight and lipid levels in HFD‐induced obese rats, and found the underlying mechanism to be associated with the regulation of intestinal flora composition. Next, we will further investigate the putative mechanism of SS against obesity and its metabolic side effects from the viewpoint of liver metabolomics.

## CONFLICT OF INTEREST

The authors have no conflicts of interest to declare.

## ETHICS STATEMENT

All animal experiments were conducted in accordance with the Affairs Concerning Experimental Animals, and they were approved by the Guizhou Medical University Animal Ethics Committee (Ethics No. 2000221).
